# Engagement of persons with dementia: how to co‐construct research?

**DOI:** 10.1002/alz70860_105153

**Published:** 2025-12-23

**Authors:** Sathya Karunananthan, Isabella Moroz, Nalia Gurgel Juarez, Krishnpriya Singh, Suey Yeung, Ngozi Faith Iroanyah, Genevieve Arsenault‐Lapierre, Rosette Loughlin, Deanne Houghton, Mary Beth Wighton, Cheryl Levi, Heidi Sveistrup, Sanjna Navani, Clare Liddy, Claire Godard‐Sebillotte, Mwali Muray

**Affiliations:** ^1^ University of Ottawa, Ottawa, ON, Canada; ^2^ Bruyere Health Research Institute, Ottawa, ON, Canada; ^3^ University of, Ottawa, ON, Canada; ^4^ Alzheimer Society of Ontario, Toronto, ON, Canada; ^5^ McGill University, Montreal, QC, Canada; ^6^ The Ottawa Hospital, Ottawa, ON, Canada; ^7^ Université d'Ottawa, Ottawa, ON, Canada

## Abstract

Social determinants of health, such as racialization and socioeconomic status, influence both the risk of developing dementia and access to quality care. However, many dementia studies lack representation from underserved communities, resulting in gaps in understanding their unique needs and the barriers they face in accessing appropriate care. With growing awareness of and interest in addressing these gaps, sharing effective engagement strategies and inclusive research practices becomes essential.

This presentation will explore some of the challenges of engaging people living with dementia from underserved communities and offer evidence‐based strategies to address these challenges. We will focus on approaches that consider individual needs and socio‐cultural contexts while ensuring that voices from diverse communities are included and valued. Drawing on our experiences with participatory research, we will share recommendations on how researchers and healthcare providers can meaningfully collaborate with members of underserved groups to co‐design inclusive research methodologies, recruitment strategies, and interventions. These approaches are crucial for improving the representation of underserved communities in dementia research and ensuring their needs are reflected in care delivery design.

As a case study, we will present our experience of co‐designing an inclusive interview guide and recruitment strategy for people living with dementia and their care partners from racially and economically diverse backgrounds to gather their perspectives on electronic consultations (eConsult) as a means of accessing specialist care. This example highlights how a participatory, co‐design approach can bridge the gap between research and underserved communities, fostering inclusivity and shared ownership of research processes and outcomes.

This session will offer an opportunity to discuss participatory and co‐design approaches as innovative methods in Alzheimer's disease and related dementias research and to foster international collaborations among researchers and patient partners to advance inclusive research practices and equity in dementia care.

